# Case Report: The second valve drag technique for managing acute total obstruction of the right coronary artery during transcatheter aortic valve replacement

**DOI:** 10.3389/fcvm.2025.1535890

**Published:** 2025-03-05

**Authors:** Yanbin Song, Xiaochun Zhang, Daxin Zhou, Wenzhi Pan

**Affiliations:** ^1^ Department of Cardiology, Wujin Hospital Affiliated with Jiangsu University, The Wujin Clinical College of Xuzhou Medical University, Changzhou, Jiangsu, China; ^2^ Department of Cardiology, Zhongshan Hospital Affiliated with Fudan University, Shanghai, China

**Keywords:** transcatheter aortic valve replacement, coronary obstruction, valve dislocation, drag technique, bicuspid aortic valve

## Abstract

**Introduction:**

Despite technological advancements and new generation devices availability, transcatheter aortic valve replacement (TAVR) for bicuspid aortic valve (BAV) stenosis still presents unique technical challenges.

**Methods and results:**

We report an uncommon but critical complication of acute right coronary artery occlusion resulting from valve dislocation during TAVR. For the first time, we employed novel approach, namely second valve dragging, to address mispositioned self-expanding valve. This implementation of novel and successful interventional treatment led to the rapid relief of coronary obstruction.

**Discussion:**

This innovative approach offers a promising avenue for further management of these patients in critical conditions.

## Case report

**Figure 1:**
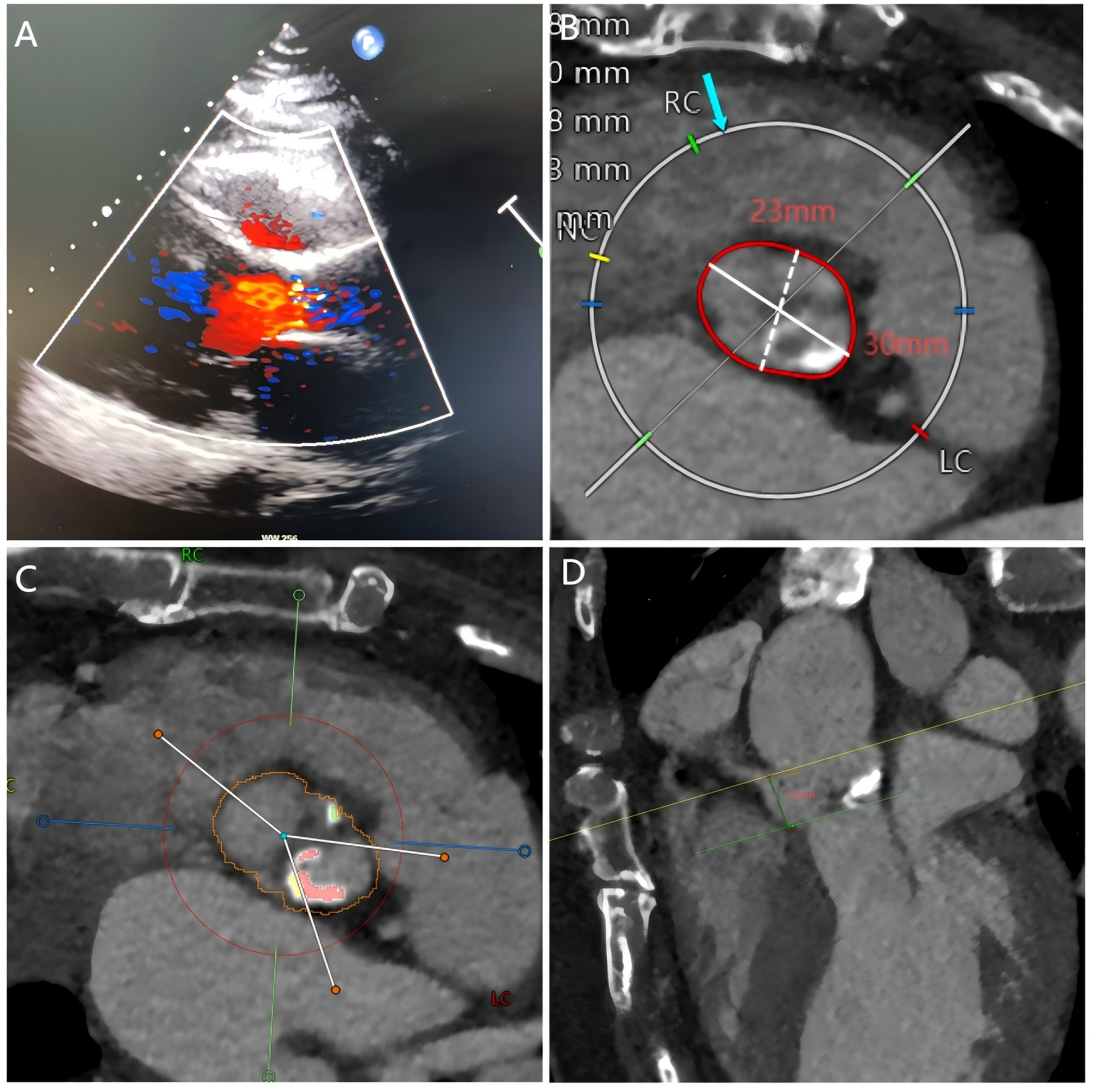
Echocardiography and cardiac computed tomography prior to TAVR. **(A)** Echocardiography showed severe aortic stenosis. **(B–D)** Pre-TAVR CT showed the aortic annulus diameter, calcified bicuspid aortic valve, and the position of the right coronary artery.

A 61-year-old female patient with a medical history of hypertension and diabetes mellitus was admitted to our institution for acute heart failure. She presented with New York Heart Association functional class III-IV and no evidence of coronary artery disease. Echocardiography examination revealed the presence of a bicuspid aortic valve (BAV) and severe aortic stenosis (AS), with peak pressure gradient of 104 mmHg and peak valve velocity of 5.1 m/s (Figure 1A). Based on the recommendation from the multidisciplinary team (MDT), transcatheter aortic valve replacement (TAVR) was chosen as the treatment option for this symptomatic and high-risk patient (EuroSCORE of 7).

**Figure 2:**
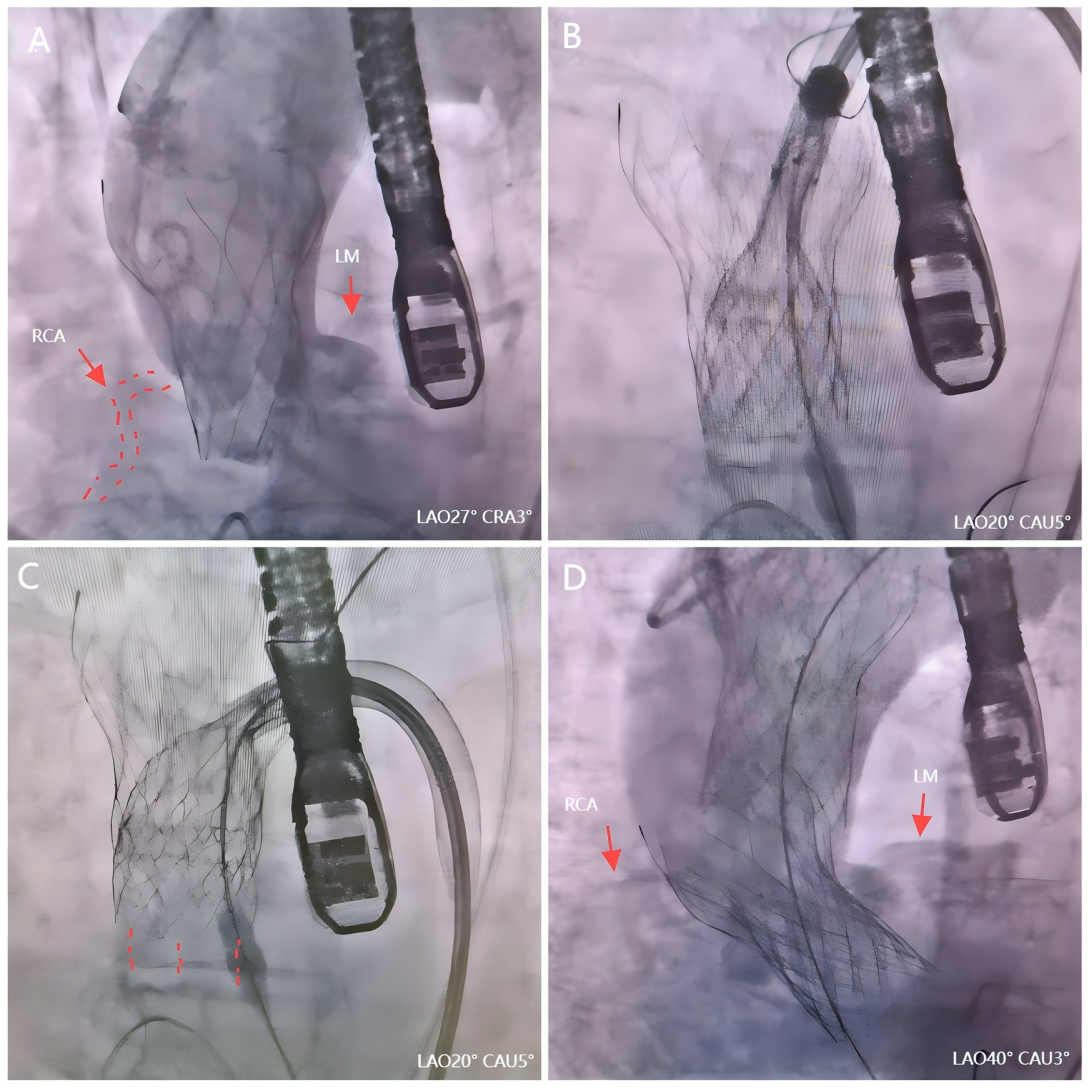
The second valve drag technique during TAVR. **(A)** Angiography revealed an acute obstruction of the right coronary artery. **(B)** The second valve successfully captured the first dislocated valve. **(C)** The first valve was repositioned by pulling the second valve toward the aorta arch. **(D)** Valve-valve intervention effectively addressed AS.

The selection of a 23 mm Taurus self-expanding valve (1) was based on pre-TAVR CT (Figures 1B–D) and transesophageal echocardiography (TEE) findings. With active root imaging assistance and pigtail catheter positioning assessment, successful deployment of the valve within the aortic annulus was achieved. Unfortunately, the valve was deployed too high, and acute obstruction of the right coronary artery (RCA) was observed through TEE and radiography subsequent to valve deployment (Figure 2A). Hypotension and pulseless electrical activity necessitated sustained cardiopulmonary resuscitation.

We firstly used one Snare from the left femoral artery to pull the valve but it failed because it caused different axis between the valve and ascending aorta. Then we tried to use the second Snare from the right radial artery to change coaxiality but it could not catch the valve stent in a short time. Several minutes after use of the second Snare, the ECG monitors showed obvious elevation of ST segment and the hemodynamics became unstable (blood pressure felled to 40/20 mmHg). Thus, we quickly switched to the second valve drag technique. A second 23 mm Taurus self-expanding valve was promptly prepared within 3 min following this severe complication. With the aid of a snare, it successfully captured the first dislocated valve by leveraging its maximum expansion capacity (Figure 2B). Then we used the second valve and its delivery system to pull up the first valve through pull the second valve toward the aorta arch (Figure 2C). Ultimately, the initial dislocated valve was safely dragged into the ascending aorta, resulting in restoration of RCA in just 7 min. The patient's circulation had also been stabilized. Subsequently, successful valve-valve intervention effectively addressed AS and paravalvular leakage (Figure 2D). During the observation period, no aortic dissection or mitral valve dysfunction were detected, with an average pressure gradient of 14 mmHg and a velocity of 1.8 m/s. Subsequently, the patient received post-TAVR treatment in the cardiac care unit (CCU).

## Comment

The persistent differences between bicuspid and tricuspid aortic stenosis (AS) can be attributed to various anatomical features, including enlarged dimensions of all components within the aortic valve complex and an increased burden of eccentric and asymmetrical calcification (2). These factors may potentially contribute to an elevated risk of valve dislocation or transcatheter aortic valve replacement (TAVR) failure. Therefore, despite technological advancements and new generation devices availability, TAVR for BAV stenosis still presents unique technical challenges. Valve dislocation, a serious complication, is associated with unacceptable clinical outcomes such as coronary artery obstruction for these patients. Coronary obstruction following TAVR is an infrequent yet potentially fatal complication. The occurrence of coronary obstruction poses a significant risk and often precipitates hemodynamic instability in affected patients. Previous treatment modalities included emergency percutaneous coronary intervention (PCI) or emergency coronary artery bypass grafting (CABG). However, the former is associated with technical complexities and a low success rate, whereas the latter necessitates extensive preparation time and surgical procedures, potentially resulting in delayed rescue for patients (3, 4). The present case reports for the first time a third approach, namely second valve dragging, which is characterized by its simplicity and rapid efficiency. In this case, right coronary artery obstruction was successfully resolved in just seven minutes. Rapid relief of coronary obstruction can reverse hemodynamic impairment and provide additional time for subsequent interventions. Furthermore, the dragging force is strong and the delivery system is less likely to ascend into the aortic arch compared to snare. Pulling the dislocated transcatheter heart valve (THV) may indeed pose a risk of ascending aorta injury, potentially leading to aortic dissection or rupture. Several key considerations are essential to minimize the additional risk of complications. Firstly, operators must have extensive experience in TAVI procedures. Secondly, by utilizing one or two snares and leveraging the maximum expansion capacity of the second THV, it is possible to successfully recapture the first dislocated valve. Thirdly, the procedure should be performed slowly and smoothly with utmost caution, while being closely monitored using radiography and transesophageal echocardiography to minimize the risk of complications.

In this case study, we emphasize the feasibility and efficacy of employing the second valve drag technique during TAVR for managing patients with mispositioned self-expanding valves and acute obstruction of the right coronary artery. This innovative approach offers a promising avenue for further management of these patients in critical conditions, particularly those facing imminent mortality, thereby addressing emergency situations more effectively.

## Data Availability

The original contributions presented in the study are included in the article/Supplementary Material, further inquiries can be directed to the corresponding authors.

## References

[1] Wang M Song G Chen M Feng Y Wang J Liu X Twelve-month outcomes of the TaurusOne valve for transcatheter aortic valve implantation in patients with severe aortic stenosis. EuroIntervention. (2022) 17:1070–6. 10.4244/EIJ-D-21-00040 34338639 PMC9725063

[2] Vincent F Ternacle J Denimal T Shen M Redfors B Delhaye C Transcatheter aortic valve replacement in bicuspid aortic valve stenosis. Circulation. (2021) 143:1043–61. 10.1161/CIRCULATIONAHA.120.048048 33683945

[3] Ribeiro HB Webb JG Makkar RR Cohen MG Kapadia SR Kodali S Predictive factors, management, and clinical outcomes of coronary obstruction following transcatheter aortic valve implantation: insights from a large multicenter registry. J Am Coll Cardiol. (2013) 62:1552–62. 10.1016/j.jacc.2013.07.040 23954337

[4] Ojeda S González-Manzanares R Jiménez-Quevedo P Piñón P Asmarats L Amat-Santos I Coronary obstruction after transcatheter aortic valve replacement: insights from the Spanish TAVI registry. JACC Cardiovasc Interv. (2023) 16:1208–17. 10.1016/j.jcin.2023.03.024 37225292

